# Effect of long-term blood pressure trajectory on the future development of chronic kidney disease: an analysis of data from the Korean National Insurance Health Checkup Study

**DOI:** 10.4178/epih.e2024090

**Published:** 2024-11-19

**Authors:** Wonmook Hwang, Eu Jin Lee, Jae-Hyeong Park, Soon-Ki Ahn

**Affiliations:** 1 Division of Cardiology, Department of Internal Medicine, Chungnam National University Sejong Hospital, Sejong, Korea; 2 Division of Nephrology, Department of Internal Medicine, Chungnam National University Hospital, Daejeon, Korea; 3 Department of Cardiology in Internal Medicine, Chungnam National University School of Medicine, Chungnam National University Hospital, Daejeon, Korea; 4 Department of Preventive Medicine, Chungnam National University Hospital, Daejeon, Korea

**Keywords:** Chronic kidney disease, Blood pressure, Trajectories, Hypertension

## Abstract

**OBJECTIVES:**

Chronic kidney disease (CKD) is a prevalent health issue that causes the irreversible loss of functioning nephrons, end-stage renal disease, cardiovascular disease, and premature mortality. Hypertension is the leading cause of CKD. However, the effect of long-term blood pressure (BP) changes on the development of CKD is still unknown. Therefore, the current study investigated the association between BP trajectory and the future development of CKD.

**METHODS:**

In this study, 246,874 individuals aged ≥40 years who underwent health examinations during the screening period (2002–2009) were evaluated. The systolic blood pressure (SBP) trajectory was determined using latent-class mixture modeling. New-onset CKD was identified during the follow-up period (2010–2019). The association between SBP trajectories and new-onset CKD was assessed.

**RESULTS:**

In total, 111,900 adults (53,420 females, 51.9±6.4 years old) presented with 2 SBP trajectory classes: class 1 (n=66,935) and class 2 (n=44,965). During the follow-up period, patients with SBP trajectory class 2 had an approximately 2.1-fold increased risk of developing CKD (unadjusted hazard ratio [HR], 2.11; 95% confidence interval [CI], 1.99 to 2.25; p<0.001). In the multivariate analysis adjusted for other significant variables, SBP trajectory class 2 was significantly associated with CKD in males (HR, 1.09; 95% CI, 1.00 to 1.19; p=0.037), but not in females (HR, 1.06; 95% CI, 0.95 to 1.18; p=0.321).

**CONCLUSIONS:**

An elevated longitudinal BP was associated with a higher incidence of CKD in male participants aged ≥40 years. Nevertheless, further studies are needed to validate the clinical significance of an elevated SBP trajectory on CKD development.

## GRAPHICAL ABSTRACT

[Fig f4-epih-46-e2024090]

## Key Message

Elevated longitudinal systolic blood pressure, as determined by trajectory, was found to be associated with an increased risk of developing chronic kidney disease in male subjects over the age of 40.

## INTRODUCTION

Chronic kidney disease (CKD) is a major health issue due to its progressive nature, which leads to the irreversible loss of functioning nephrons, end-stage renal disease (ESRD), cardiovascular disease, and/or premature mortality [1]. Common causes of CKD include hypertension, diabetes mellitus (DM), and glomerulonephritis. Its prevalence in middle-income and high-income countries has risen to approximately 10%, driven by the increasing prevalence of these etiological factors and an aging population. CKD is strongly linked to increased morbidity and mortality related to cardiovascular disease. Consequently, there is a growing medical and social imperative to predict declines in kidney function to prevent the adverse outcomes associated with CKD.

Among the common risk factors for CKD, hypertension is the most significant. In the United States, about 20% of patients with hypertension also have CKD [2]. Hypertension can lead to kidney injury through several mechanisms, such as activation of the renin-angiotensin-aldosterone system (RAAS), glomerular hypertension, endothelial dysfunction, and increased oxidative stress, all of which contribute to nephrosclerosis [3,4].

Based on 2 large-population cohort studies, elevated blood pressure (BP) is an independent risk factor for CKD and ESRD in patients with hypertension who do not have preexisting CKD [5,6]. Instead of evaluating the long-term effects of hypertension over an extended period, these studies focused on the presence or absence of hypertension at a specific time point as a variable.

Recently, there has been growing interest in the impact of BP trajectories, or the longitudinal patterns of BP, on cardiovascular diseases [7–9]. Results indicated that participants with higher BP trajectories over a 25-year follow-up period faced an increased risk of coronary artery calcifications during middle age [7]. Another cohort study demonstrated that the 10-year BP trajectories of middle-aged males could predict cardiovascular mortality. This study also utilized the decile distribution ratio of the household insurance premium to show that the longitudinal effects of BP might help identify hypertensive-mediated organ damage (HMOD) in real-world settings.

CKD is among the most common types of HMOD. Consequently, it is hypothesized that BP trajectories may influence the progression of CKD. In a prior study involving 1,837 CKD patients, an increasing systolic blood pressure (SBP) trajectory was linked to a 1.28-fold higher risk of adverse kidney outcomes compared to a stable SBP trajectory [10]. However, to date, evidence regarding the impact of BP trajectory on CKD development in non-CKD populations remains limited. Therefore, this study aimed to evaluate the effect of SBP trajectory on the future development of CKD in middle-aged individuals undergoing routine health checkups.

## MATERIALS AND METHODS

### Data source

A population-based cohort dataset from the Korean National Health Insurance Service-National Sample Cohort 2.0 was analyzed. This dataset, which represents approximately 2% of the total Korean population in 2006, includes both retrospective and prospective follow-up data spanning from 2002 to 2019. The Korean government stratified the total population into 2,142 strata based on age, sex, region, eligibility status, and income level, and then randomly selected individuals from each stratum to include in the cohort (n=1,021,208, 2.1%) [11]. Given that the Korean National Health Insurance Service covers about 97% of the total Korean population, this cohort accurately represents the entire Korean population. The cohort consists of 4 databases: (1) medical insurance eligibility, which includes socioeconomic variables of the participants, type of health insurance, and records of birth and death; (2) medical treatments, encompassing medical bills claimed by medical service providers and diagnoses based on the International Classification of Diseases, 10th revision (ICD-10) codes; (3) information about the medical care institution, such as the type of institution, its establishment, and location; and (4) nationwide general health examinations of the cohort members. The Korean government advises that all Korean adults undergo a basic health checkup every 2 years. This checkup includes completing questionnaires on medical history and health-related behaviors, undergoing simple chest radiography and anthropometric measurement with BP, and having blood tests. Additionally, the cohort includes mortality data sourced from the death registration database of Statistics Korea, the central government organization responsible for statistics.

### Study population

This study screened adults aged ≥40 years who participated in national health checkups and underwent more than 1 national health examination between 2002 and 2009. The BP trajectory was determined using the SBP values recorded during biannual health checkups up to 2009. The prespecified exclusion criteria included: (1) individuals previously diagnosed with CKD at the time of screening, (2) those who died during the screening period, and (3) those with fewer than 3 BP readings during the study period.

### Outcomes

The primary outcome of this study was the incidence of CKD during the follow-up period from 2009 to 2019, categorized by BP trajectory groups. CKD was defined as the first occurrence, on at least 2 different days, of hospital visits, admission for CKD, or procedures related to CKD, including hemodialysis. CKD identification utilized medical claims data. In this context, a CKD diagnosis required the first occurrence during at least 2 different days of outpatient hospital visits or during the initial admission, suggesting a probable CKD diagnosis. Diagnoses were determined using ICD-10 codes, specifically N18, N18.1, N18.2, N18.3, N18.4, N18.5, N18.9, Z49, Z94, Z94.0, O7020, O7021, O7031, O7032, O7033, O7034, and R3280.

Data were censored at the time of incident CKD or at the end of the study (December 31, 2019).

Hypertension was defined as an SBP ≥140 mmHg and/or diastolic blood pressure (DBP) ≥90 mmHg or the use of antihypertensive medication based on the questionnaire responses. Participants with a fasting plasma glucose level ≥126 mg/dL or those who were treated with insulin or oral antidiabetic drugs were considered to have DM. Baseline cigarette smoking was classified into 3 groups: never smokers, previous smokers, and current smokers. Baseline alcohol consumption frequency was categorized into 3 groups: none, 1–2, and ≥3 times/wk. The baseline volume of alcohol consumption was classified into 3 groups: 0, <140, and ≥140 g/wk. Baseline physical activity was categorized into 3 groups: none, 1–4, and ≥5 day/wk. Baseline exercise intensity was expressed as metabolic equivalents (METs) and classified into 3 groups: <500, 500–1,000, and >1,000 METs. Using the decile distribution ratio of the household insurance premium, the family income status was categorized as follows: low (deciles 1–3), middle (deciles 4–7), and high (deciles 8–10). Trained personnel measured the height and weight of the participants according to the written protocol. The body mass index (BMI) was calculated by dividing weight (kg) by height (m^2^). The baseline blood tests included fasting glucose, total cholesterol, and hemoglobin levels.

### Classification of systolic blood pressure trajectories

BP values from the right upper arm, collected during a nationwide health checkup, were used in this study. The SBP values necessary for calculating the BP trajectory were selected from participants who had more than 3 BP measurements over an 8-year period in the current dataset. Latent-class mixed modeling (LCMM) was applied to identify distinct subpopulations within the larger population, characterized by similar developmental SBP trajectories over time. These models were fitted using the LCMM package (version 2.0.2) in R version 4.3.0 following a 3-step procedure [7,12,13]. The LCMM package models longitudinal data as a discrete mixture of 2 or more latent trajectories through maximum likelihood [9,12,13]. The analysis of SBP trajectories using LCMM was conducted through a multistep process. Initially, we hypothesized that SBP trajectories could be categorized into 2–4 distinct groups. During the model development phase, a censored normal model was employed to fit the trajectories, acknowledging the continuous nature of SBP. The modeling began with a linear polynomial and involved progressively comparing the model fit using the Bayesian information criterion (BIC) across different numbers of trajectories. A lower BIC value indicates a better model fit, effectively balancing model complexity with the goodness-of-fit to the sample data. In the final step of model selection, the optimal model was chosen based on several criteria: (1) the lowest BIC value, which balances model complexity and goodness-of-fit; (2) average posterior probabilities of group membership exceeding 0.6 for individuals in each group; (3) odds of correct classification based on posterior probabilities surpassing a threshold of 5; (4) each trajectory group comprising more than 5% of the sample to ensure a distinct distribution; and (5) clinical relevance of the identified trajectories.

Using this methodological approach, we conducted a comprehensive analysis of the SBP trajectories, combining statistical rigor with clinical relevance. The chosen model offered the most parsimonious representation of the data, while still being practically relevant to patterns in cardiovascular health [14,15].

Finally, the best fitting model, based on the criteria mentioned above, was the linear trajectories of 2 groups. Supplementary Materials 1–3 provide the statistical information for various models.

Based on the modeling process, 2 distinct SBP trajectory groups were identified. The first group, designated as SBP trajectory class 1, included individuals who maintained relatively low BP levels. The second group, designated as SBP trajectory class 2, comprised individuals who maintained relatively high BP levels (Supplementary Material 4).

### Statistical analysis

The characteristics of the participants were presented as means with standard deviations or as counts and percentages. To identify statistically significant differences between the 2 groups, we employed the Student t-test or the chi-square test. We conducted multivariate analysis using the Cox proportional hazards model to evaluate the associations between SBP trajectories and the development of CKD. Hazard ratios (HRs) and 95% confidence intervals (CIs) were calculated. All analyses were carried out using SAS version 9.2 (SAS Institute Inc., Cary, NC, USA) and R version 4.3.0 (R Foundation for Statistical Computing).

### Ethics statement

The study protocol received approval from the Institutional Review Board of the Chungnam National University Hospital (approval No. CNUH 2024-01-050). This research adhered to the most recent version (2013) of the World Medical Association’s Declaration of Helsinki, which outlines the code of ethics for research involving human subjects. This study used a sample cohort from the Korean National Health Checkup. The institutional review board waived the need for informed consent.

## RESULTS

### Characteristics of participants according to systolic blood pressure trajectories

**Figure 1:**
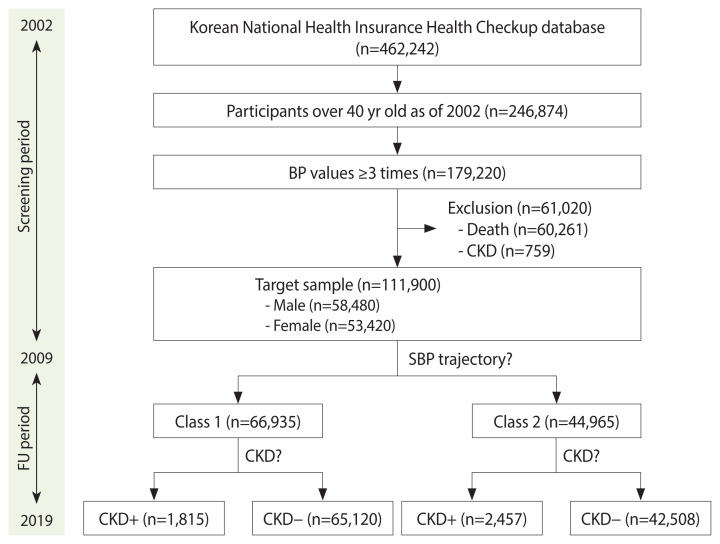
Selection of participants. BP, blood pressure; CKD, chronic kidney disease; SBP, systolic blood pressure; FU, follow-up.

Figure 1 provides an overview of participant selection. The study included adults aged 40 years and older who underwent a national health examination more than once between 2002 and 2009. A total of 61,020 participants who either died or were diagnosed with CKD during the study period were excluded. Subsequently, the SBP trajectory pattern was assessed for 111,900 participants (53,420 females), each of whom had at least 3 BP readings. Over the 8-year period, the distribution of BP readings among participants was as follows: 48,024 (42.8%) had 3 readings, 37,397 (33.4%) had 4, 8,040 (7.2%) had 5, 6,091 (5.4%) had 6, 5,436 (4.9%) had 7, and 6,912 (6.2%) had 8 readings. The average number of BP readings per participant in this study was 4.1±1.4.

### Characteristics of participants according to systolic blood pressure trajectories

**Figure 2:**
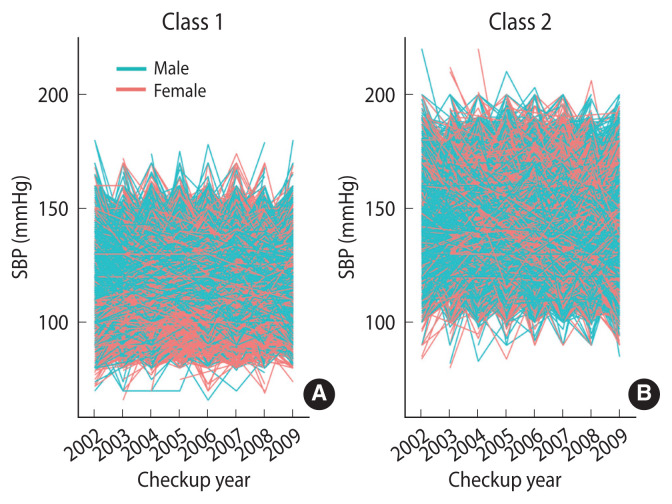
Systolic blood pressure (SBP) trajectory groups (A) class 1 and (B) class 2 over time during the study period.

The participants were categorized into 2 groups: class I (n=66,935), referred to as the normal SBP trajectory group, and class 2 (n= 44,965), known as the elevated SBP trajectory group (Figure 2, Supplementary Material 4). The normal SBP trajectory group comprised a significantly higher proportion of female participants than the elevated SBP trajectory group. Additionally, this group exhibited a significantly lower prevalence of cardiovascular risk factors such as hypertension, DM, and current smoking than the elevated SBP trajectory group (Table 1). Members of the normal SBP trajectory group were notably younger and had significantly lower levels of SBP, DBP, BMI, fasting blood glucose, total cholesterol, and hemoglobin compared to those in the elevated SBP trajectory group. However, there were no significant differences between the 2 groups in terms of alcohol consumption frequency and income status.

### Characteristics of the participants with chronic kidney disease

During the follow-up period, 4,272 participants were diagnosed with CKD (Figure 1). Table 2 presents the characteristics of the participants with CKD. The CKD group included a significantly higher proportion of males compared to the group without CKD. Additionally, the CKD group had a significantly higher prevalence of cardiovascular risk factors, including hypertension, DM, and current smoking, than the group without CKD. Furthermore, the CKD group exhibited significantly higher levels of BMI, SBP, DBP, fasting blood glucose, total cholesterol, and hemoglobin compared to the group without CKD.

### Systolic blood pressure trajectories and the future development of chronic kidney disease

**Figure 3:**
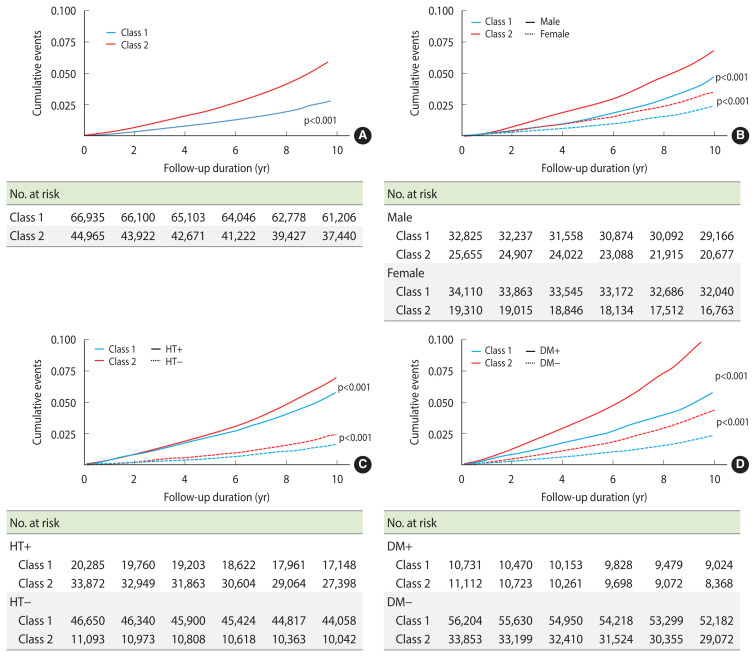
Incidence rate of new-onset chronic kidney disease (CKD) according to the systolic blood pressure (SBP) trajectory groups in the entire cohort based on the Kaplan–Meier analysis (A), SBP trajectory class 2 represents an increased incidence of CKD independent of sex (B), presence of hypertension (HT) (C), and presence of diabetes mellitus (DM) (D).

Table 3 shows the association between SBP trajectories and the subsequent development of CKD. The group with elevated SBP trajectories exhibited approximately a 2.1-fold higher risk of developing CKD compared to the group with normal SBP trajectories (unadjusted HR, 2.11; 95% CI, 1.99 to 2.25; p<0.001). Figure 3 presents the development of CKD over time, as visualized using a Kaplan–Meier curve. The incidence of CKD was higher in the elevated SBP trajectory group than in the normal SBP trajectory group. Furthermore, an elevated SBP trajectory was linked to the development of CKD, regardless of sex or the presence of hypertension and diabetes (Figure 3).

SBP trajectory class 2 was significantly associated with CKD after adjusting for age and sex (model I: adjusted HR, 1.52; 95% CI, 1.43 to 1.62; p<0.001) and additional clinical variables (model II: adjusted HR, 1.07; 95% CI, 1.00 to 1.15; p=0.035). In the multivariate analysis, which was adjusted for other significant variables, SBP trajectory class 2 showed a significant association with CKD in male participants (HR, 1.09; 95% CI, 1.00 to 1.19; p=0.037); however, this association was not observed in female participants (HR, 1.06; 95% CI, 0.95 to 1.18; p=0.321).

## DISCUSSION

This study showed that a higher SBP trajectory (class 2) was positively associated with the development of CKD in individuals aged ≥40 years. After performing multivariate analysis adjusted for other clinical variables, a higher SBP trajectory was significantly associated with CKD in male patients.

High BP can narrow or constrict the renal blood vessels, reducing renal blood flow and worsening renal function. BP is an independent risk factor for kidney outcomes [16]. Both SBP and DBP are linked to cardiovascular disease-related mortality and damage to target organs. However, elevated SBP significantly influences the progression of CKD [17,18]. Arterial stiffness, a primary cause of systolic hypertension, is associated with a decreased glomerular filtration rate and impaired kidney function due to mechanisms such as endothelial dysfunction, collagen accumulation, and inflammation in smooth muscle cells [19,20]. Therefore, individuals with high BP are at an increased risk of developing CKD.

However, BP values fluctuate over time, and their longitudinal pattern can be described as a trajectory [9]. These BP trajectories consolidate longitudinal BP patterns into a single metric that accounts for both the absolute BP levels and the rate of BP changes over time. Elevated BP trajectories may indicate prolonged exposure to high BP levels and are associated with an increased risk of cardiovascular events linked to hypertension.

In 11,181 Chinese participants aged >60 years, an elevated SBP trajectory was associated with increased mortality (HR, 1.34; 95% CI, 1.23 to 1.45) [21]. The SBP trajectory also provided the best incremental discriminative value for cardiovascular events compared to traditional risk profiles in a prospective community-based cohort study in China [22].

A high-increasing SBP trajectory from childhood to young adulthood was associated with an increased left ventricular mass index [23]. Kim et al. [24] recently reported that higher mid-BP trajectories were an independent risk factor for HMOD in middle-aged individuals. The study demonstrated that modeling mid-BP trajectories can be effective in identifying high-risk individuals with multiple BP readings in the era of smart electronic medical records. However, further research is needed to explore the relationship between BP trajectories and the development of HMOD.

Increasing evidence has revealed a clear association between temporal BP trajectories and adverse kidney outcomes in patients with CKD [10]. Joo et al. [25] reported that an increased SBP trajectory was significantly associated with adverse kidney outcomes, based on prospective cohort data (n=1,837). This study indicated that patients in the increased SBP trajectory group had a 1.28-fold higher risk of ESRD compared to those with stable BP trajectories. However, groups with stable or decreased BP trajectories did not exhibit a high-risk of ESRD. Another study demonstrated that an increased SBP trajectory, ranging from 105 mmHg to 124 mmHg, was significantly associated with the development of new-onset CKD in a cohort of 4,643 participants without CKD and hypertension, as part of the Korean Genome and Epidemiology Study [25].

Our data revealed that the presence of hypertension and DM at baseline can increase the risk of developing CKD. Hypertension can lead to the deterioration of kidney function through the induction of nephrosclerosis. This condition results from the activation of the RAAS, increased glomerular hypertension, endothelial dysfunction, and heightened oxidative stress. The baseline kidney function was not assessed in this study. However, it is possible that patients with hypertension had diminished kidney function at the outset. In patients with hypertension who presented with early-stage CKD, elevated BP may have exacerbated the decline in already compromised kidney function [26].

DM is a well-established risk factor for CKD, with patients suffering from DM facing a significantly higher risk of adverse kidney outcomes. Chronic hyperglycemia in these patients leads to progressive damage to the kidney’s microvasculature, ultimately resulting in renal dysfunction [27]. Those with poorly controlled blood sugar levels and unmanaged hypertension are particularly at an increased risk of developing ESRD [28]. Furthermore, chronic inflammation and insulin resistance in DM patients can cause an excessive accumulation of extracellular matrix proteins within the renal tissues, exacerbating kidney damage [29].

In this study, an increased SBP trajectory was found to be an independent predictor of CKD in men only. While male and female patients showed significant differences across several variables (Supplementary Material 5), SBP trajectory class 2 significantly influenced the onset of new CKD solely in male patients. Our findings align with those from other research. For instance, a systematic review and meta-analysis involving 2,382,712 individuals indicated that females had a 23% lower risk of developing CKD or ESRD compared to males (relative risk, 0.77; 95% CI, 0.63 to 0.95) [30]. However, the underlying reasons for this sex disparity remain unclear. The differential impact of BP on CKD development in males could potentially be attributed to sex differences, varying hormonal effects, and the severity of cardiovascular risk factors.

This study evaluated the impact of longitudinal BP on the future development of CKD but faced several limitations. Firstly, incident CKD was identified solely using ICD-10 codes, which may have led to misdiagnoses and misclassifications of CKD. Secondly, there was no data available on baseline serum creatinine levels during the screening period, as this was not included in the routine examinations mandated by the Korean government at that time. Consequently, it was not possible to confirm the number of individuals with high serum creatinine levels at baseline or to establish a statistically significant difference between the 2 groups. Thirdly, the study relied on variables from routine health examinations and did not adjust for other variables that would incur additional costs or require further testing. Fourthly, as a cohort study, it could suggest a causal relationship between elevated SBP trajectories and increased CKD incidence. However, the inherent limitations of observational studies mean that some factors influencing CKD development might not have been identified. Prospective studies with comprehensive data are needed to clarify this potential causal relationship. Fifthly, the analysis focused exclusively on SBP trajectories, without considering other relevant factors such as DBP, pulse pressure (SBP-DBP), and BP variability in relation to CKD development. Future research should explore the impact of these BP parameters on CKD progression. Sixthly, the study included only participants who had undergone regular health checkups on more than 3 occasions, which may introduce selection bias. Finally, the study population consisted solely of Korean individuals, limiting the generalizability of the findings to other ethnic groups.

In conclusion, an elevated longitudinal BP assessed using the SBP trajectory was associated with an increased risk of new-onset CKD in participants aged over 40 years. This association remained statistically significant even after adjusting for other variables and was observed exclusively in males. To establish a causal relationship between an increased SBP trajectory and incident CKD, further studies with larger sample sizes and more precise diagnostic criteria are needed.

### Future perspectives

Elevated BP is a well-known risk factor for cardiovascular diseases, including CKD. This study demonstrated that tracking the BP trajectory during the screening period can help identify individuals at high-risk of developing incident CKD. Therefore, monitoring SBP trajectories could be beneficial in high-risk cohorts undergoing routine health checkups to prospectively detect new-onset CKD. This research could offer opportunities to prevent future CKD events. Additionally, patients with CKD in our cohort showed differences in sex distribution. However, further research is needed to explore the mechanisms behind this sex difference.

## Figures and Tables

**Figure f4-epih-46-e2024090:**
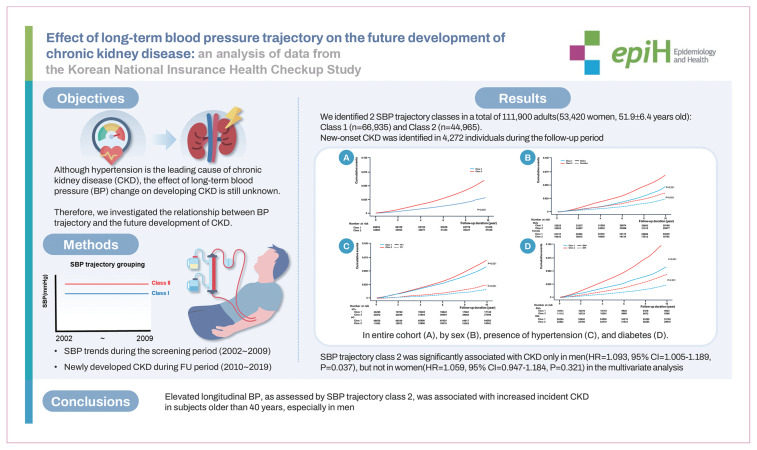


**Table 1:** Participants’ characteristics according to SBP trajectory groups

Characteristics	SBP trajectory		p-value
	Class 1 (n=66,935)	Class 2 (n=44,965)	
Sex, female	34,110 (51.0)	19,310 (42.9)	<0.001
Age (yr)	50.0±8.2	54.6±9.2	<0.001
40–44	21,151 (31.6)	7,372 (16.4)	
45–54	27,669 (41.3)	15,862 (35.3)	
55–64	13,513 (20.2)	14,434 (32.1)	
≥65	4,602 (6.9)	7,297 (16.2)	
Body mass index (kg/m^2^)	23.5±2.8	24.7±2.9	<0.001
Cigarette smoking			<0.001
Never smoker	45,065 (69.0)	29,542 (67.3)	
Past smoker	5,850 (9.0)	4,583 (10.4)	
Current smoker	14,440 (22.1)	9,758 (22.2)	
Alcohol drinking (times/wk)			
Never drinker	39,214 (59.6)	24,074 (54.5)	
1–2	20,934 (31.8)	14,041 (31.8)	
≥3	5,670 (8.6)	6,063 (13.7)	
Alcohol drinking amount (g/wk)			<0.001
None	24,231 (62.3)	14,916 (57.9)	
<140	9,379 (24.1)	5,845 (22.7)	
≥140	5,309 (13.6)	5,011 (19.4)	
Exercise (day/wk)			<0.001
None	36,218 (55.6)	24,358 (55.7)	
1–4	22,830 (35.1)	14,595 (33.4)	
≥5	6,065 (9.3)	4,788 (10.9)	
Exercise strength (METs)			<0.001
<500	26,555 (68.2)	17,470 (67.7)	
500–1,000	8,512 (21.9)	5,490 (21.3)	
>1,000	3,884 (9.97)	2,859 (11.1)	
Status of income			0.112
Low income	14,530 (22.0)	10,444 (23.6)	
Middle income	22,407 (34.0)	15,789 (35.6)	
High income	29,055 (44.0)	18,115 (40.8)	
SBP (baseline, mmHg)	117.0±12.3	140.0±15.1	<0.001
DBP (baseline, mmHg)	74.5±9.4	86.2±10.3	<0.001
CKD	1,815 (2.7)	2,457 (5.5)	<0.001
Hypertension	20,285 (30.3)	33,872 (75.3)	<0.001
Diabetes mellitus	10,731 (16.0)	11,112 (24.7)	<0.001
Baseline chemical profiles			
Fasting glucose (mg/dL)	93.8±20.0	99.1±24.3	<0.001
Total cholesterol (mg/dL)	197.0±36.7	204.0±38.1	<0.001
Hemoglobin (g/dL)	13.8±1.5	14.1±1.5	<0.001

Values are presented as mean±standard deviation or number (%).

SBP, systolic blood pressure; METs, metabolic equivalents; DBP, diastolic blood pressure; CKD, chronic kidney disease.

**Table 2:** Participants’ characteristics according to CKD

Characteristics	CKD–(n=107,628)	CKD+ (n=4,272)	p-value
Sex, female	55,783 (48.2)	2,697 (36.9)	<0.001
Age (yr)	51.6±8.8	57.5±9.0	<0.001
40–44	28,100 (26.1)	423 (9.9)	
45–54	42,350 (39.3)	1,181 (27.6)	
55–64	26,304 (24.4)	1,643 (38.5)	
≥65	10874 (10.1)	1,025 (24.0)	
Body mass index (kg/m^2^)	23.4±2.9	24.6±3.0	<0.001
Cigarette smoking			<0.001
Never smoker	71,947 (66.8)	2,660 (62.3)	
Past smoker	9,964 (9.3)	469 (11.0)	
Current smoker	23,173 (21.5)	1,025 (24.0)	
Alcohol drinking (times/wk)			<0.001
Never drinker	60,878 (56.6)	2,410 (56.4)	
1–2	33,739 (31.3)	1,236 (28.9)	
≥3	11,189 (10.4)	544 (12.7)	
Alcohol drinking amount (g/wk)			0.013
None	37,658 (96.2)	1,489 (3.8)	
<140	14,724 (96.7)	500 (3.3)	
≥140	9,952 (96.4)	368 (3.6)	
Exercise (day/wk)			<0.001
None	58,251 (54.1)	2,325 (54.4)	
1–4	36,118 (33.6)	1,307 (30.6)	
≥5	10,325 (9.6)	528 (12.4)	
Exercise strength (METs)			0.147
<500	42,386 (96.3)	1,639 (3.7)	
500–1,000	13,530 (96.6)	472 (3.4)	
>1,000	6,493 (96.3)	250 (3.7)	
Status of income			0.112
Low income	24,045 (22.3)	929 (21.7)	
Middle income	36,780 (34.2)	1,416 (33.1)	
High income	45,304 (42.1)	1,866 (43.7)	
SBP (baseline, mmHg)	126.0±17.0	133.0±18.6	<0.001
DBP (baseline, mmHg)	79.1±11.3	81.8±11.4	<0.001
SBP trajectory class			<0.001
Class 1	65,120 (60.5)	1,815 (42.5)	
Class 2	42,508 (39.5)	2,457 (57.5)	
Hypertension	50,854 (47.2)	3,303 (77.3)	<0.001
Diabetes mellitus	20,192 (18.8)	1,651 (38.6)	<0.001
Baseline chemical profiles			
Fasting glucose (mg/dL)	95.6±21.5	105.0±31.3	<0.001
Total cholesterol (mg/dL)	200.0±37.3	203.0±40.5	<0.001
Hemoglobin (g/dL)	13.9±1.5	14.0±1.5	<0.001

Values are presented as mean±standard deviation or number (%).

CKD, chronic kidney disease; METs, metabolic equivalents; SBP, systolic blood pressure; DBP, diastolic blood pressure.

**Table 3:** Association between systolic blood pressure trajectories and the presence of chronic kidney disease

Variables	Unadjusted	p-value	Model I^1^	p-value	Model II^2^	p-value
Total (n=111,900)						
Class 1 (n=66,935)	1.00 (reference)		1.00 (reference)		1.00 (reference)	
Class 2 (n=44,965)	2.11 (1.99, 2.25)	<0.001	1.52 (1.43, 1.62)	<0.001	1.07 (1.00, 1.15)	0.035
Male (n=58,480)						
Class 1 (n=32,825)	1.00 (reference)		1.00 (reference)		1.00 (reference)	
Class 2 (n=25,655)	2.01 (1.86, 2.17)	<0.001	1.56 (1.45, 1.69)	<0.001	1.09 (1.00, 1.19)	0.037
Female (n=53,420)						
Class 1 (n=34,110)	1.00 (reference)		1.00 (reference)		1.00 (reference)	
Class 2 (n=19,310)	2.10 (1.91, 2.32)	<0.001	1.47 (1.33, 1.64)	<0.001	1.06 (0.95, 1.18)	0.321

Values are presented as hazard ratio (95% confidence interval).

1 Model I: Adjusted for sex, age (age groups, 4 categories).

2 Model II: Adjusted for sex, age (age groups, 4 categories), body mass index (kg/m^2^), fasting glucose (mg/dL), hemoglobin (g/dL), cigarette smoking (3 categories), past history of hypertension, past history of diabetes mellitus.
