# Supplementary material for: Effect of long-term blood pressure trajectory on the future development of chronic kidney disease: an analysis of data from the Korean National Insurance Health Checkup Study
Source: Epidemiol Health. 2024 Nov 19;46:e2024090. doi: 10.4178/epih.e2024090 (PMC11840418; doi:10.4178/epih.e2024090)
Supplement: Supplementary file 1 [file epih-46-e2024090-Supplementary.docx]

**Supplementary Material 1. Model fit evaluation information for each LCMM tested**

|  | Maximum log-likelihood | BIC |
| --- | --- | --- |
| **Two-class LCMM** | **-1891400** | **3782869.51** |
| Three-class LCMM | -1891833 | 3783770.14 |
| Four-class LCMM | -1892048 | 3784235.90 |

BIC, Bayesian information criteria; LCMM, latent class mixed model

The best fitting model is highlighted in bold characters**.**

**Supplementary Material 2. Posterior probabilities in each LCMM tested**

|  | Class 1 | Class 2 | Class 3 | Class 4 |
| --- | --- | --- | --- | --- |
| **Two-class LCMM** | **0.6881** | **0.7292** |  |  |
| Three-class LCMM | 0.3793 | 0.4284 | 0.3407 |  |
| Four-class LCMM | 0.2634 | 0.251 | 0.2889 | 0.2551 |

LCMM, latent class mixed model

The best fitting model is highlighted in bold characters.

**Supplementary Material 3. Posterior classification in each LCMM tested**

|  | Class 1 | Class 2 | Class 3 | Class 4 |
| --- | --- | --- | --- | --- |
| **Two-class LCMM** | **59.82%** | **40.18%** |  |  |
| Three-class LCMM | 65.15% | 27.39% | 7.46% |  |
| Four-class LCMM | 66.18% | 1.07% | 29.34% | 3.41% |

LCMM, latent class mixed model

The best fitting model is highlighted in bold characters.

**Supplementary Material 4**

Trend of blood pressure according to the systolic blood pressure (SBP) trajectory group during the screening period


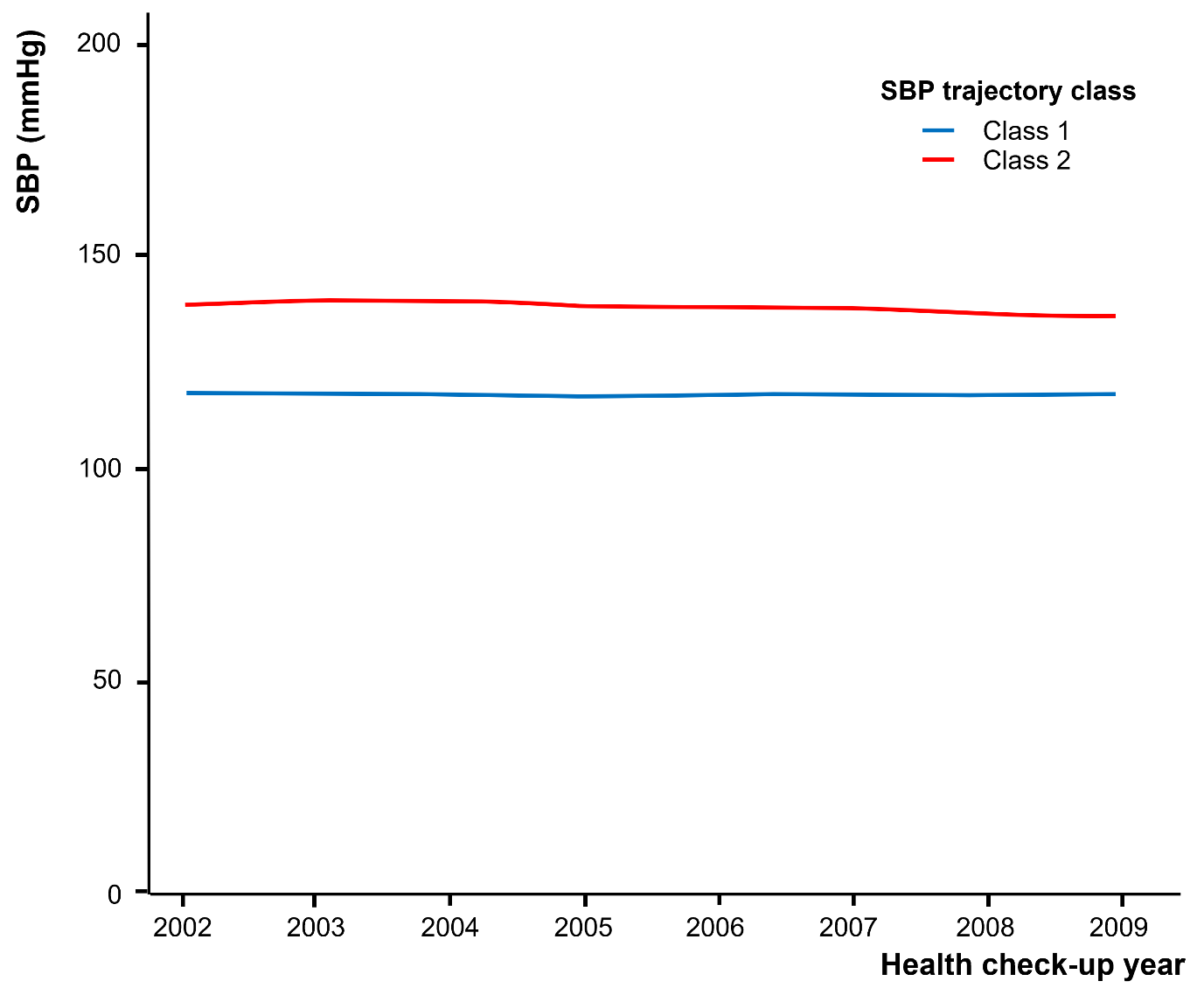


**Supplementary Material 5. Participants’ characteristics according to sex**

|  | Female  (n=53,420) | Male  (n=58480) | *P* value |
| --- | --- | --- | --- |
| SBP trajectory class |  |  | <0.001 |
| Class 1 | 34,110 (63.9%) | 32,825 (56.1%) |  |
| Class 2 | 19,310 (36.1%) | 25,655 (43.9%) |  |
| Age (years old) | 52.4 ± 9.0 | 51.4 ± 8.8 | <0.001 |
| Age groups |  |  | <0.001 |
| 40-44 years old  45-54 years old  55-64 years old  ≥ 65 years old | 12,369 (23.2%)  20,694 (38.7%)  14,134 (26.5%)  6,223 (11.6%) | 16,154 (27.6%)  22,837 (39.1%)  13,813 (23.6%)  5,676 (9.7%) |  |
| BMI (kg/m^2^) | 24.0 ± 3.0 | 24.0 ± 2.8 | <0.001 |
| Cigarette smoking |  |  | <0.001 |
| Never smoker (%) | 50,063 (97.0%) | 24,544 (42.6%) |  |
| Past smoker (%) | 455 (0.9%) | 9,978 (17.3%) |  |
| Current smoker (%) | 1,119 (2.2%) | 23,079 (40.1%) |  |
| Alcohol drinking |  |  | <0.001 |
| Never drinker (%) | 43,077 (82.5%) | 20,217 (35.0%) |  |
| 1-2 times/week (%) | 8,277 (15.8%) | 26,682 (46.2%) |  |
| ≥3 times/week (%) | 882 (1.7%) | 10,863 (18.8%) |  |
| Exercise |  |  | <0.001 |
| None (%) | 33,295 (64.1%) | 27,281 (48.0%) |  |
| 1-4 days/week (%) | 13,574 (26.1%) | 23,851 (41.9%) |  |
| ≥5 days/week (%) | 5,098 (9.8%) | 5,753 (10.1%) |  |
| Status of income |  |  | <0.001 |
| Low income | 13,199 (25.0%) | 11,775 (20.5%) |  |
| Middle income | 18,292 (34.6%) | 19,904 (34.6%) |  |
| High income | 21,332 (40.4%) | 25,838 (44.9%) |  |
| SBP (baseline, mmHg) | 124.5 ± 18.0 | 128.1 ± 16.6 | <0.001 |
| DBP (baseline, mmHg) | 77.3 ± 11.4 | 80.9 ± 11.0 | <0.001 |
| CKD | 1,575 (3.0%) | 2,697 (4.6%) | <0.001 |
| Hypertension (%) | 26,229 (49.2%) | 27,858 (47.6%) | <0.001 |
| Diabetes mellitus (%) | 10,400 (19.5%) | 11,443 (19.6%) | 0.682 |
| Baseline chemical profiles |  |  |  |
| Fasting glucose (mg/dL) | 93.8 ± 19.8 | 97.9 ± 23.6 | <0.001 |
| Total cholesterol (mg/dL) | 201.9 ± 38.1 | 198.1 ± 36.7 | <0.001 |
| Hemoglobin (g/dL) | 12.9 ± 1.2 | 14.8 ± 1.1 | <0.001 |

Data were expressed as mean ± SD or number of people (%).

BMI: body mass index; BP: blood pressure; BUN: blood urea nitrogen; CRP: C-reactive protein; DBP: diastolic blood pressure; DM: diabetes mellitus; SBP: systolic blood pressure
